# Analysis of Emergency Department Utilization in Medicaid Expansion and Non-expansion States

**DOI:** 10.7759/cureus.18561

**Published:** 2021-10-07

**Authors:** Ashley Lall, Randolph Devereaux, Mike Flynn, Christian Vandever, Krystal Tomsky-Jackson

**Affiliations:** 1 Emergency Medicine, Mercer University School of Medicine, Macon, USA; 2 Public Health, Edward Via College of Osteopathic Medicine, Monroe, USA; 3 Public Health, Hospital Corporation of America, Charleston, USA; 4 Statistics, Hospital Corporation of America, Brentwood, USA; 5 Statistics, Coliseum Medical Centers, Macon, USA

**Keywords:** medicaid expansion, ambulatory care sensitive conditions, emergency department utilization, insurance status, affordable care act

## Abstract

Introduction

The Affordable Care Act has been debated since its initial enactment over a decade ago. One of the primary topics for discussion has been Medicaid expansion, which has created a schism across the United States. The effects of Medicaid expansion largely remain unclear. The purpose of this report is to elucidate how Medicaid expansion has impacted emergency department (ED) utilization by analyzing Medicaid expansion and non-expansion states to determine who visited the ED and the reason for the visit.

Methods

We conducted a retrospective analysis using de-identified electronic medical record (EMR) data from 56,423 patients and 33 different hospitals (18 Medicaid non-expansion and 15 Medicaid expansion) who visited the ED in 2019. We used geographical demographics and insurance status to categorize patients who visited the ED and ambulatory care sensitive conditions (ACSC) to identify the reasons for the visit. Logistic regression and chi-square analysis were used to analyze the data.

Results

We observed a significant relationship between Medicaid expansion and geographic region such that patients living in rural or semirural regions likely resided in Medicaid non-expansion states. Patients using self-pay were more likely to live in a Medicaid non-expansion state than a Medicaid expansion state (32.3% vs. 21.5%, p-value < 0.0001). Finally, there were no significant differences between the top five ACSC for Medicaid expansion and Medicaid non-expansion states but living in an expansion state was significantly (p < 0.01) related to being diagnosed with an ACSC (OR, 1.056; 95% CI, 1.013-1.100).

Conclusion

In conclusion, Medicaid expansion was associated with differences in the use of medical resources. Patients using Medicaid insurance who reside in Medicaid expansion states preferentially use the ED. Geographical location does play a role in ED utilization and ambulatory care sensitive condition diagnoses in patients. Despite these findings, the full effects of Medicaid expansion on ED utilization require further investigation. However, our research indicates that Medicaid expansion is not the singular solution in decreasing ED utilization and healthcare costs.

## Introduction

On March 23, 2010, the Affordable Care Act became law with the goal of extending healthcare coverage [1]. An unprecedented effect was the emergence of coverage disparities causing 36 US states to expand Medicaid, while 14 states chose not to expand Medicaid [2]. The effects of this legislation on emergency department (ED) visits remain unclear. Nikpay et al. found that emergency department visits increased significantly in states with larger increases in Medicaid enrollment. Before the expansion, Kentucky had one of the lowest Medicaid eligibility guidelines for adults, but following expansion, there were a large number of childless adults newly eligible for Medicaid leading to an increase in ED visits. In contrast, states with previous Medicaid coverage for childless adults saw little change in the number of ED visits [3]. Furthermore, a national analysis revealed that 8% of the patients responsible for the increase in ED visits repeatedly come to the ED with non-emergent complaints, accounting for 28% of all ED visits [4,5]. These patients may also be classified as having ambulatory care sensitive conditions (ACSC), which may be better treated with primary care interventions instead of in the ED [6-8]. One study found that 77% of adults visited the ED because of the severity of their illness, 12% reported their doctor’s office was not open, and 7% stated lack of access to a primary care physician. Patients with Medicaid were more likely to report the reason for their ED visit was because of the seriousness of their illness, while uninsured patients were more likely to use the ED because of lack of access to care [9].

Navigating the best methods to provide quality patient care while keeping healthcare costs as low as possible is a difficult balance for hospital administrators. The balance is made more difficult when the potential influences of the new legislation are not carefully considered. Therefore, the purpose of this study was to analyze the differences in ED utilization between Medicaid non-expansion and Medicaid expansion states, specifically evaluating the ambulatory care sensitive conditions requiring the ED visit. With this information, our goal was to improve our understanding of Medicaid expansion healthcare legislation and its effects on ED use. We also wanted to identify factors that bring patients to the ED to prevent increased ED utilization in the future.

## Materials and methods

This was a retrospective analysis of the Hospital Corporation of America (HCA) Healthcare Data Warehouse, using electronic medical record (EMR) data from 18 Medicaid non-expansion state hospitals in Georgia (Cartersville Medical Center, Coliseum Medical Centers, Doctors Hospital of Augusta, and Fairview Park Hospital) and Tennessee (Centennial Medical Center, Centennial Medical Center - Ashland City, Eastside Medical Center, Hendersonville Medical Center, Horizon Medical Center, Parkridge East Hospital, Parkridge Medical Center, Parkridge West Hospital, Redmond Regional Medical Center, Skyline Medical Center, Southern Hills Medical Center, Stonecrest Medical Center, Summit Medical Center, and Tulane University Hospital & Clinic), which provided records from 30,895 patients who fit our criteria. For Medicaid expansion states, we chose 15 hospitals in Kentucky (Frankfort Regional Medical Center and Greenview Regional Hospital), Louisiana (Lakeview Regional Medical Center and Rapides Regional Medical Center), and Virginia (CJW Medical Center, Henrico Doctors Hospital, John Randolph Medical Center, LewisGale Hospital - Alleghany, LewisGale Hospital - Montgomery, LewisGale Hospital - Pulaski, LewisGale Medical Center, Memorial Satilla Health, Reston Hospital Center, Spotsylvania Regional Medical Center, and StoneSprings Hospital Center), which provided 25,528 patients who fit our criteria.

Inclusion criteria were patients 18 to 64 years of age who were uninsured, had Medicaid insurance, or private third-party insurance. The patients’ records were de-identified and obtained from a 12-month period during 2019. To account for seasonal fluctuation of ED visits, we examined the records on the 1st and 15th days of each month over the 12-month period. Patient records were grouped by gender, insurance status, and Environmental Systems Research Institute (ESRI) demographics [10].

After the appropriate patient records were obtained, we selected the following ambulatory care sensitive conditions (ACSC) to be included in the analysis: angina, asthma, bacterial pneumonia, cellulitis, diabetes complications, congestive heart failure (CHF), chronic obstructive pulmonary disease (COPD), dehydration, gastroenteritis, epileptic convulsion, hypertension (HTN), hypoglycemia, kidney urinary infection, nutrition deficient, severe ear, nose, and throat (ENT) infection, anxiety disorder, opioid adverse reaction, panic disorder, alcohol dependence, and major depressive disorder. The International Classification of Diseases, Tenth Revision (ICD-10) codes for each ACSC listed were used to determine what illness brought our patients to the ED.

Logistic regression was conducted for the ambulatory care sensitive conditions (ACSC), with Medicaid expansion status, gender, and geographic location included as covariates. Additionally, a chi-square analysis of ESRI categories (rural, semirural, suburban periphery, metro cities, urban periphery, and principal urban center) between Medicaid expansion and Medicaid non-expansion groups was conducted. Chi-square analysis was also used for the evaluation of the relationship between expansion status and payer type. An independent t-test (unequal variance) was used to compare the top five ASCS (hypertension, diabetes complications, asthma, COPD, and severe ENT infections) in Medicaid expansion and Medicaid non-expansion states. Finally, we performed a sub-analysis of two, similarly sized, hospitals with different Medicaid expansion statuses using a chi-square analysis.

## Results

There were a total of 56,423 patients who visited the ED in 2019 at 33 different hospitals, largely in the mid-Atlantic and Southeast US included in our study. Each patient’s geographical demographic (ESRI urbanization), insurance status, and reason for the visit were determined. A chi-square analysis of ESRI urbanization between Medicaid expansion and Medicaid non-expansion (Table 1) allowed us to determine that patients who visited the ED were more likely to live in a Medicaid non-expansion state than a Medicaid expansion state (25.7% vs. 17.8%, p-value <0.0001) if they resided in a rural area.

**Table 1:** Counts (column %) for emergency department visits for hospitals in Medicaid non-expansion and expansion states based on ESRI urbanization category. p < 0.0001, chi-square analysis. ESRI, Environmental Systems Research Institute.

	Medicaid non-expansion	Medicaid expansion	Total
Rural (n)	5542	3018	8560
	25.7%	17.8%	22.2%
Semirural (n)	2832	1451	4283
	13.2%	8.5%	11.1%
Suburban periphery (n)	4063	5087	9150
	18.9%	30.0%	23.8%
Metro cities (n)	4571	4077	8648
	21.2%	24.0%	22.5%
Urban periphery (n)	4321	3045	7366
	20.1%	17.9%	19.1%
Principal urban center (n)	197	297	494
	0.9%	1.7%	1.3%

Logistic regression was performed to determine the relationships between the binary variable ambulatory care sensitive conditions (ACSC) and Medicaid expansion status, ESRI urbanization, and sex (Table 2). Only 16,776 patients (of 56,423) were included in our selected list of ICD-10 codes for ACSC. Nevertheless, we were able to determine that patients living in expansion states had higher odds (5.6%) of having an ambulatory care sensitive condition diagnosis compared to patients living in non-expansion states (OR, 1.056; CI, 1.013-1.100, p < 0.01). In addition, male patients were 1.123 times more likely to have an ACSC diagnosis when compared to female patients (p < 0.0001). Lastly, for the logistic regression analysis, we found that patients residing in principal urban centers and suburban peripheries were 26% and 20% less likely to have an ACSC diagnosis than patients residing in rural areas (Table 2) (p < 0.015 and p < 0.0001, respectively). A bivariate analysis of the top five ACSC diagnoses for Medicaid expansion and Medicaid non-expansion did not reveal a statistical difference (p = 0.62).

**Table 2:** Logistic regression for ambulatory care sensitive conditions. * = statistically significant.

	Significance	Exp (B)	95% confidence interval (lower, upper)
Expansion	0.01*	1.056	1.013, 1.100
Gender	0.0001*	1.123	1.077, 1.171
Principal urban center	0.015*	0.794	0.660, 0.956
Urban periphery	0.894	1.004	0.943, 1.069
Metro cities	0.923	0.997	0.939, 1.059
Suburban periphery	0.0001*	0.833	0.784, 0.844
Semirural	0.627	0.982	0.912, 1.057

When analyzing payer status for each patient’s ED visit, chi-square analysis revealed a significant relationship between expansion status and payer type (Table 3). Patients who visited the ED using self-pay as their form of insurance identification were more likely to live in a Medicaid non-expansion state than a Medicaid expansion state (32.3% vs. 21.5%, p-value < 0.0001).

**Table 3:** Counts (column %) for emergency department visits for hospitals in Medicaid non-expansion and expansion states based on payer source. p < 0.0001, chi-square analysis.

	Medicaid non-expansion	Medicaid expansion	Total
Third-party	11249	11755	23004
	36.4%	46.0%	40.8%
Medicaid	9667	8291	17958
	31.3%	32.5%	31.8%
Self-pay	9979	5482	15461
	32.3%	21.5%	27.4%

Our sub-analysis of a 328-bed hospital in a Medicaid expansion state (Louisiana), compared to a 310-bed hospital in a Medicaid non-expansion state (Georgia) yielded a similar outcome (Table 4). Patients who visited the ED at the Louisiana hospital were more likely to be insured through Medicaid than patients who visited the ED at the Georgia hospital (64% vs. 30.6%, p < 0.0001, respectively). Patients who visited the Louisiana hospital ED were less likely to be self-pay (12.6%), compared with those who visited the Georgia hospital ED (34.1%, p < 0.0001).

**Table 4:** Counts (column %) for emergency department visits from a sub-analysis comparing a Georgia hospital (Medicaid non-expansion) to a Louisiana hospital (Medicaid expansion). p < 0.0001, chi-square analysis.

	Georgia hospital (Medicaid non-expansion)	Louisiana hospital (Medicaid expansion)	Total
Third-party	1024	628	1652
	35.3%	23.4%	29.6%
Medicaid	890	1716	2606
	30.6%	64.0%	46.7%
Self-pay	990	338	1328
	34.1%	12.6%	23.8%

## Discussion

Our analyses allowed us to evaluate who was using the ED, the reasons for the ED visits, and whether there were differences between states with Medicaid expansion or Medicaid non-expansion. It is important to note that our study analysis is retrospective and thus relied on EMR data inputted at the time of each patient's visit. These data did not include all the demographic variables used in the study for every patient that fit our inclusion criteria. In addition, the specificity of our ICD-10 diagnoses that were selected may have excluded other important ACSC that brought patients to the ED. Keeping these limitations in mind, we were able to produce meaningful results and answer our key objectives.

With respect to our first objective, we determined that patients who visited the ED from rural areas were more likely to live in Medicaid non-expansion states (Table 1). In general, ED visits in rural areas are largely increased when compared to ED visits in urban areas. When ED visits from 2005 to 2016 were reviewed by Greenwood-Ericksen and Kocher, they found that rural ED visit rates increased over that time period from 36.5 to 64.5 visits per 100 persons. Over the same time period, urban ED visits did not change substantially (40.2 to 42.8 per 100 persons). In fact, by 2016, one-fifth of all ED visits were in rural areas [11]. To provide a better understanding of the ESRI rural categorization, these individuals are likely to live in an area with less than 50 people per square mile, are the least ethnically diverse, and are likely to be working blue-collar jobs, be self-employed, or retired [10]. ED visits have been reported to be generally increased in rural areas. We found substantially higher ED use in rural and semirural areas in Medicaid non-expansion vs. Medicaid expansion states. Foutz, Artiga, and Garfield also reported that rural areas with Medicaid expansion had substantially increased Medicaid coverage rates compared to non-expansion areas, leading to higher rates of non-elderly uninsured in these regions [12]. Although most US rural counties located in the South and Midwest include states that chose not to expand Medicaid, when we analyzed our raw data, ED visits in Medicaid non-expansion states had fewer total visits (25,528) when compared to Medicaid expansion states (30,895). Thus, the increase of ED utilization in these rural areas found in our study was not due to the general trend of increase rural ED use nationwide.

To evaluate what brought patients to the ED, we analyzed the ambulatory care sensitive conditions trends. We found that the top five ACSC (hypertension, diabetes complications, asthma, COPD, and severe ENT infections) were not statistically different between Medicaid non-expansion states and Medicaid expansion states. A study from 2011, which analyzed the top five reasons for ED visits by age, revealed the most common reason for patients 18 to 44 years of age and 45 to 64 years of age to be sprains/strains and non-specific chest pain, respectively. Other top reasons for each age group included headache, abdominal pain, otitis media, and intervertebral disc disorder [13]. It is important to note that in our study, we selected specific ambulatory care sensitive conditions for analysis. Therefore, the differences in the top five ACSC in our study compared to the aforementioned study is related to these selections. For example, we did not search for the ICD-10 code for “chest pain" but used the ICD-10 code for "angina." However, what is the most important takeaway from our study is that despite controlling for Medicaid expansion, our top ambulatory care sensitive conditions for ED visits were the same in both Medicaid non-expansion and Medicaid expansion states.

We also found that patients living in expansion states had significantly higher odds of having an ambulatory care sensitive condition diagnosis (Table 2). To understand what this information might mean in a broader context, we must ask how Medicaid non-expansion states had more visits to the ED in a 12-month period but were less likely to have an ambulatory care sensitive condition diagnosis. We suspect the answer is multifactorial and requires an improved understanding of the definition of ambulatory care sensitive condition, which is “any condition that could be prevented through intervention in the primary care setting” [6]. We would expect that in a population with more health insurance coverage, there would be a lower frequency of ACSC diagnoses in the ED; however, Gingold et al. did not observe a decrease in ACSC-related visits after Medicaid expansion. These researchers analyzed ED utilizers and ED visits both six months prior to Medicaid expansion in Maryland and 12 months after expansion. They determined that there was a slight increase in ED utilization after expansion. Additionally, Gingold et al. reported that ACSC-related visits among high ED utilizers, which was defined as greater than four ED visits in six months, were not decreased following Medicaid expansion [14,15]. We found that patients in Medicaid expansion states were more likely to have an ACSC diagnosis, leading us to conclude that simply being insured does not alter patients’ preferential use of primary care in outpatient settings. It is possible that our time frame (12 months) was not sufficient to effectively assess Medicaid expansion and the potential impact on ambulatory care sensitive conditions.

We found that patients living in principal urban centers and suburban periphery were less likely to have an ACSC diagnosis than those residing in rural areas (Table 2). Principal urban centers are described as diverse populations of 2.5 million in density. Suburban peripheries are typically affluent suburbs and account for one-third of the US population [10]. A study from Korea in 2019 found that higher numbers of primary care providers were correlated with decreased ACSC hospitalization rates. They also used ACSC hospitalizations as measurements for access to primary care, which means that there is an inverse relationship between these variables [16]. With this in mind, we acknowledge that our patients living in principal urban centers and suburban peripheries also live in areas with more available primary care resources and thus have less ambulatory care sensitive conditions [17]. However, this is likely an oversimplification as it is difficult to determine the link between geographical locations and primary care availability and how much this impacts a patient’s decision to go to the ED. Other considerations, such as health literacy, transportation, individual motivation, and financial responsibilities, create an intricate interplay between healthcare access and geographic locations that goes beyond the number of primary care resources [18].

Finally, we observed that patients who visited the ED using self-pay were more likely to live in a Medicaid non-expansion state (Table 3). When analyzing our data, third-party insurance status accounted for the majority of all ED visits in both Medicaid non-expansion (11,249) and Medicaid expansion states (11,755) while self-pay accounted for fewer ED visits (9,979 vs. 5,482, respectively). It has been shown nationally that ED visits are highest for patients with Medicaid and lowest for patients with private insurance [19]. This followed in our sub-analysis of two like-sized hospitals in urban areas of the southeast (Louisiana and Georgia) with different expansion statuses (Table 4). A study of Maryland post-Medicaid expansion found that Medicaid-covered ED visits increased by 5.6% while uninsured patient ED visits decreased by 5.9%. However, this change in insurance coverage did not increase ED volume, meaning that the same cohort of patients who previously were self-pay was now eligible for Medicaid [20].

## Conclusions

Ultimately, our data lead us to suggest that patients using Medicaid insurance who also reside in Medicaid expansion states will continue to preferentially use the ED. Patients from Medicaid non-expansion states who visit the ED are more likely to be self-pay. Geographical location does play a role in ED utilization and ambulatory care sensitive condition diagnoses in patients. This calls into question how access to care and health literacy interplay in a patient’s choice to use a primary care facility. Our findings suggest that Medicaid expansion does not appear to be the singular solution to reducing ED visits and healthcare costs. Most likely, the solution requires increased health and insurance literacy, which may be better obtained through tailored community health programs rather than large, federal legislative changes.
